# Evaluation of circulating small extracellular vesicle-derived miRNAs as diagnostic biomarkers for differentiating between different pathological types of early lung cancer

**DOI:** 10.1038/s41598-022-22194-0

**Published:** 2022-10-13

**Authors:** Yi-fang Jiang, Shan-na Wei, Nan Geng, Wen-wen Qin, Xin He, Xiu-huan Wang, Yao-pu Qi, Shan Song, Ping Wang

**Affiliations:** Department of Respiratory Medicine, Fourth Hospital of Hebei Medical University, Shijiazhuang, 050011 People’s Republic of China

**Keywords:** Lung cancer, Evolution

## Abstract

Lung cancer is the leading cause of cancer-related death worldwide. MicroRNAs (miRNAs) in circulating small extracellular vesicles (sEVs) have been suggested to be potential biomarkers for cancer diagnosis. The present study was designed to explore whether plasma-derived sEV miRNAs could be utilized as diagnostic biomarkers for differentiating between early-stage small cell lung cancer (SCLC) and early-stage non-small cell lung cancer (NSCLC). We compared the miRNA profiles of plasma-derived sEVs from healthy individuals, patients with early-stage SCLC and patients with early-stage NSCLC. Next-generation sequencing was used to screen for differentially expressed miRNAs (DEMs). Gene ontology (GO) and Kyoto Encyclopedia of Genes and Genomes (KEGG) pathway analyses were used to predict the potential functions of these DEMs. Weighted gene coexpression network analysis (WGCNA) was used to identify the different pathology-related miRNA modules. We found that 22 DEMs were significantly different among healthy individuals, patients with early-stage SCLC, and patients with early-stage NSCLC. We selected six representative DEMs for validation by qRT‒PCR, which confirmed that miRNA-483-3p derived from plasma sEVs could be used as a potential biomarker for the diagnosis of early-stage SCLC, miRNA-152-3p and miRNA-1277-5p could be used for the diagnosis of early-stage NSCLC respectively.

## Introduction

The international cancer research centre GLOBOCAN 2020 released data on the incidence, mortality and development trends of 36 types of cancer in 185 countries/regions in December 2020. That year, nearly 2,200,000 new lung cancer cases and 1,790,000 lung cancer-related deaths were recorded. Among all tumours, the incidence of lung cancer ranked second, and its mortality rate ranked first^1^. Lung cancer patients usually experience no symptoms in early stages^2^, so the majority of patients are diagnosed at an intermediate-to-advanced stage. With the increasingly widespread adoption of follow-up routine chest computed tomography examination, the identification of patients with early lung lesions is becoming more common^3^. However, for various reasons, obtaining pathological information about some patients in the early stage remains difficult. Thus, the development of noninvasive liquid biopsy techniques for the diagnosis of early-stage lung cancer is particularly important. According to pathology, lung cancer can be divided into small cell lung cancer (SCLC) and non-small cell lung cancer (NSCLC), which have different clinical features and are treated diffrently^4^. Therefore, the differential diagnosis of these different pathological types of early-stage lung cancer is particularly important.

MicroRNAs (miRNAs) are small noncoding RNAs that are 21–23 nt in length^5^. They play important roles in many biological processes, including cell development, proliferation, differentiation and apoptosis^6^. MiRNAs have been identified in many biofluids, such as plasma, serum, and urine, suggesting the potential to develop circulating miRNAs as minimally invasive biomarkers of cancer and other diseases^7–9^. Several studies have shown that miRNAs are involved in the occurrence and development of lung cancer and that they are diagnostic and predictive biomarkers for lung cancer^10,11^.

Extracellular vesicles (EVs) are nanosized membranous vesicles released from most kinds of cells^12–14^. On the basis of their biogenesis, release pathways, and size, EVs are classified as exosomes (also called small EVs), microvesicles (MVs), and apoptotic bodies^12,13^. EVs carry bioactive cellular components such as proteins, lipids, metabolites, and nucleic acids ^15,16^. These can be transferred from the host to recipient cells and affect the function of the recipient cell ^17,18^. Recent studies have verified that sEVs are present in a variety of body fluids, such as blood, urine, cerebrospinal fluid, pleural effusion, bile, amniotic fluid and ascites, suggesting that they participate in many physiological activities^19–24^. Small EVs (sEVs) derived from the plasma of patients with cancer have been shown to play a critical role in tumorigenesis, tumour growth, metastasis and response to therapy^25–27^. The miRNAs in peripheral blood sEVs are more stable, sensitive and accurate than miRNAs in plasma, are easier to obtain than tissue materials, and could be promising novel cancer biomarkers^28^.

To date, there have been few studies on the systematic screening of plasma-derived sEV miRNAs that are related to the diagnosis of lung cancer and particularly to the differential diagnosis of different pathological types of lung cancer. In the present study, sEVs were isolated from plasma samples, RNA-seq was performed, and differentially expressed miRNAs (DEMs) of healthy individuals, patients with SCLC and patients with NSCLC were analysed. Gene ontology (GO) and Kyoto Encyclopedia of Genes and Genomes (KEGG) pathway analyses were used to identify the functions of DEMs and to search for target miRNAs in patients with SCLC and patients with NSCLC, respectively.

With the development of bioinformatics, new and efficient methods have been developed to achieve a more comprehensive identification of new biomarkers. Weighted gene coexpression network analysis (WGCNA), a systematic method aimed at identifying coexpressed genes by calculating gene connectivity^29^, was applied to construct a gene interaction network using all the detectable miRNAs. More importantly, WGCNA is an effective way to explore the mechanisms underlying certain traits^29^. However, no study has identified critical modules and genes in early-stage lung cancer using the WGCNA method. Herein, we used WGCNA for the first time to identify key modules related to early-stage SCLC and NSCLC, and discovered the different biological processes and pathways of their occurrence.

**Figure 1:**
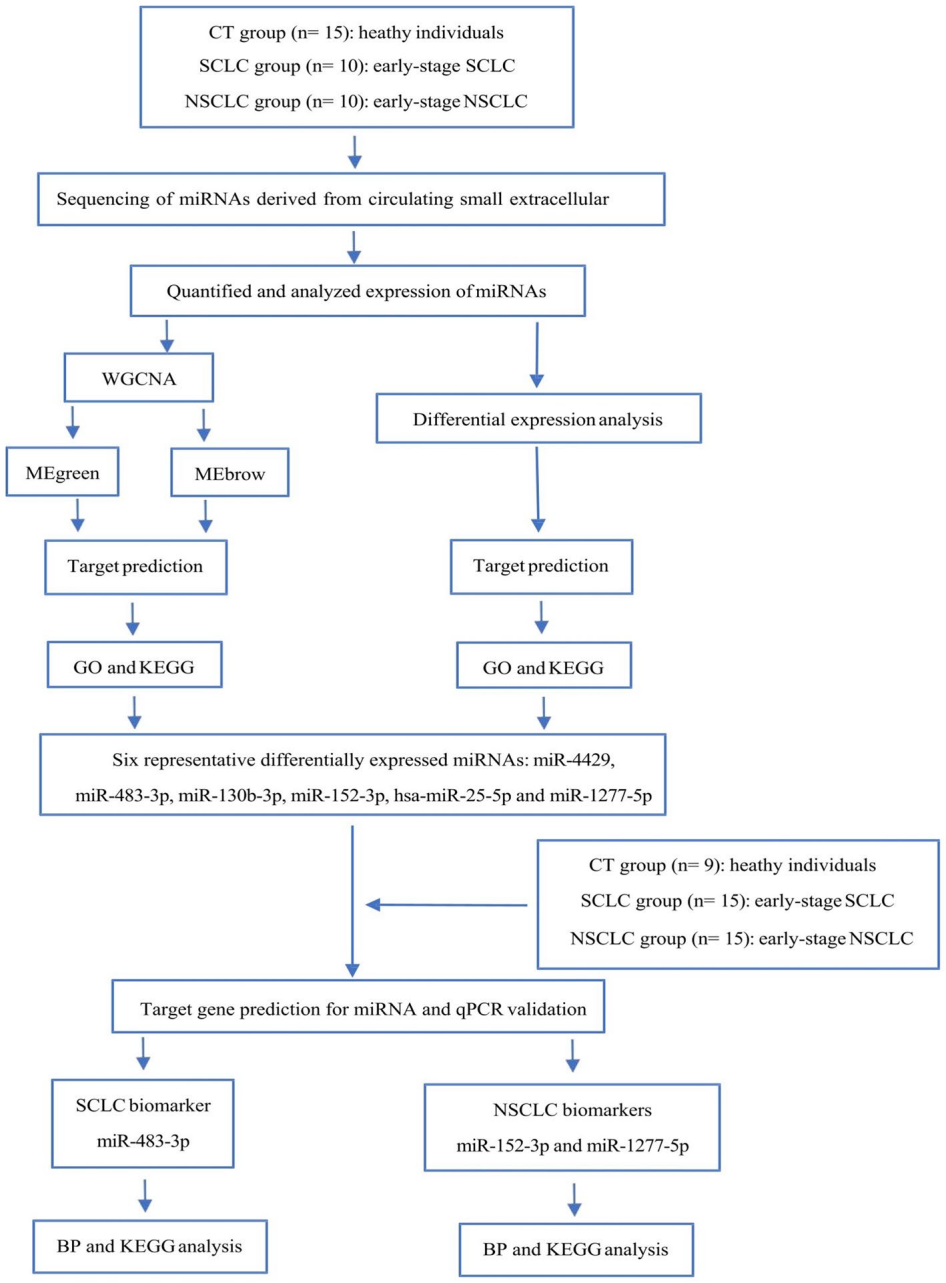
Flow chart of the study.

Finally, six representative DEMs were selected and further validated in additional healthy individuals, SCLC patients and NSCLC patients using qRT‒PCR. The aim was to explore the use of plasma-derived sEV DEMs as potential biomarkers, and these DEMS might be used as specific biomarkers for the diagnosis of early-stage SCLC and NSCLC. The steps of the experimental protocol are shown in Fig. 1.

## Material and methods

### Patient information and sample collection

A total of 35 participants, including 15 healthy individuals, 10 patients with early-stage SCLC and 10 patients with early-stage NSCLC, were enrolled in this study between August 2020 and August 2021 at the Fourth Hospital of Hebei Medical University. All the participants were subjected to miRNA sequencing. Additionally, 9 healthy individuals, 15 patients with early-stage SCLC and 15 patients with early-stage NSCLC were enrolled for qRT‒PCR validation. The inclusion criteria for lung cancer were as follows: (1) initially pathological diagnosed with no distant metastatic SCLC or NSCLC (stage I to II) and diagnosed by pathology; (2) no history of treatment; and (3) availability of peripheral blood samples. The exclusion criteria were as follows: (1) diagnosis with distant metastasis and (2) history of any other malignant disease. The TNM (T, tumour size; N, lymph node metastasis; M, distant metastasis) classification of lung cancer was restaged according to the National Comprehensive Cancer Network (NCCN) Guidelines (Version 2.2018)^4^. The healthy individuals were enrolled during physical examination that were performed during the same period of time. The inclusion criteria for healthy individuals were as follows: (1) no history of any disease; (2) no history of drug administration within the preceding three months; and (3) availability of peripheral blood samples. All the patients and healthy volunteers provided written informed consent for the collection of plasma samples and for their pathological data to be used in this study. The local medical ethics committee approved this study (Fourth Hospital of Hebei Medical University Hospital, Shijiazhuang, China; reference number 2021196). All the experiments were performed in accordance with relevant named guidelines and regulations. Blood samples were collected from patients before treatment in 4 mL vacutainer tubes with EDTA anticoagulant. After the blood samples were centrifuged at 1500 × *g* for 10 min at 4 °C and then 3000 × *g* for 15 min at 4 °C, the plasma was aspirated and stored at − 80 °C before use.

### Isolation of plasma sEVs

Small EVs were isolated from plasma using size exclusion chromatography (SEC). One millilitre of 0.8 μm-filtered blood plasma was diluted 1.5-fold with PBS and further purified using Exosupur columns (Echobiotech, China). The samples were then eluted with 0.01 M PBS, and 2 mL eluate fractions (fractions 3, 4, 5, and 6) were collected according to the manufacturer’s instructions. The fractions were concentrated to 200 μL by 100 kDa molecular weight cut-off Amicon Ultra spin filters (Merck, Germany).

### Nanoparticle tracking analysis (NTA)

Vesicle suspensions at concentrations between 1 × 10^7^/mL and 1 × 10^9^/mL were examined using a ZetaView PMX 110 (Particle Metrix, Meerbusch, Germany) equipped with a 405 nm laser. The size and quantity of the isolated particles were determined. A total of 20 μL sEVs for analysis. A video of 60-s duration was taken with a frame rate of 30 frames/sec, and particle movement was analysed using NTA software (ZetaView 8.02.28).

### Transmission electron microscopy (TEM)

A total of 10 µL sEV-enriched supernatant was placed on a copper mesh and incubated at room temperature for 10 min. The sEVs were treated with uranyl acetate solution for 1 min after washing with sterile distilled water. The sample was then dried for 2 min under incandescent light. The copper mesh was observed and photographed under a transmission electron microscope (H-7650, Hitachi Ltd., Tokyo, Japan).

### Western blot analysis

Small EV-enriched supernatant was denatured in 5 × sodium dodecyl sulfonate (SDS) buffer and subjected to Western blotting (10% SDS–polyacrylamide gel electrophoresis; 50 µg protein/lane) using rabbit polyclonal antibodies against CD63 (sc-5275, Santa Cruz, CA, USA), CD9 (60232-I-Ig, Proteintech, Rosemont, IL), TSG101 (sc-13611, Santa Cruz) and calnexin (10,427–2-AP, Promega, Madison, WI). The proteins were visualized on a Tanon4600 Automatic chemiluminescence image analysis system (Tanon, Shanghai, China).

### RNA isolation and qRT‒PCR

Total RNA was extracted from sEVs using miRNeasy Plasma Advanced Kits (Qiagen, cat. No. 217204) according to the manufacturer’s protocol. Total RNA was then reverse transcribed to synthesize cDNA using the PrimeScript™ RT reagent Kit (Perfect Real Time) (Takara, RR037A). Target gene expression was measured by TaqMan® probe using real-time qPCR. Two microlitres of cDNA was used as the template for each PCR. Then, to validate the selected miRNAs, we carried out a qPCR assay. The sequences of the primers and probes are shown in Table S1. U6 was used as an internal reference. The gene expression levels were calculated with the 2 ^—△△CT^ method.

### Library preparation and sequencing

For small RNA libraries, a total amount of 10 ng RNA per sample was used as input material for the RNA sample preparations. Sequencing libraries were generated using QIAseq miRNA Library Kits (Qiagen, Frederick, MD) following the manufacturer’s recommendations, and index codes were added to attribute sequences to each sample. Finally, the library quality was assessed on the Agilent Bioanalyzer 2100. Clustering of the index-coded samples was performed on an acBot Cluster Generation System using TruSeq PE Cluster Kitv3-cBot-HS (Illumina, San Diego, CA, USA) according to the manufacturer’s instructions. After cluster generation, the library preparations were sequenced on an Illumina HiSeq platform, and paired-end reads were generated.

### Quantification of miRNA expression and differential expression analysis

Bowtie tools software, The Clean Reads with the Silva database, GtRNAdb database, Rfam database and Repbase database sequence alignment, filter ribosomal RNA (rRNA), transfer RNA (tRNA), small nuclear RNA (snRNA), small nucleolar RNA (snoRNA) and other ncRNA and repeats were used. The remaining reads were used to identify known miRNAs and new miRNAs that were predicted by comparison with known miRNAs from miRBase and Human Genome (GRCh38), respectively. The read count for each miRNA was obtained from the mapping results, and TPM was calculated. Circulating exosomal miRNA profiles of samples with two conditions were compared by Edge R software, and each miRNA with a log2-fold change > 0.58 and *p* ≤ 0.05 was considered differentially expressed. Hierarchical clustering was performed with the R package ‘pheatmap’.

### GO and KEGG pathway enrichment analysis

The target miRNAs of the DEMs were predicted using miRanda and RNAhybrid software and filtered based on free energy and score. The screening criteria for the Miranda database were set to score ≥ 150 and energy ≤ − 20. The screening criteria for the RNAhybrid database were set to energy ≤ -25 and *p* value < 0.05. Venn analysis was applied to intersect the data obtained from the two databases. The miRNAs shared by the two databases were recognized as target miRNAs. GO analysis was used to analyse the key functions of the target genes according to Gene Ontology. Similarly, pathway analysis was performed to analyse the significant pathways of the targeted genes based on KEGG. Fisher’s exact test and χ^2^ test were performed to select the significant GO terms and pathways, and the threshold of significance was filtered by *P* value < 0.05. The validated miRNAs in enriched biological pathways were selected to construct miRNA‒target-pathway networks with Cytoscape software (Version: 3.5.1).

### WGCNA

The miRNA coexpression networks were constructed by the WGCNA package based on miRNAs. The WGCNA package (version 1.61) was used to identify lung cancer-related modules according to a previous description. In brief, a scale-free topology criterion was used to calculate the soft threshold. The optimal soft threshold was selected, and the minimum module size was set to 30 genes. The modules were identified using the dynamic tree cut, and the MEDissThres parameter was set to 0.381. After the modules were correlated with clinical features, the modules with a Pearson correlation coefficient > 0.8 were selected and merged to obtain the target module.

### Statistical analysis and bioinformatics analysis

Logistic regression analysis was used to construct models consisting of a group of candidate diagnostic miRNAs. Receiver operating characteristic (ROC) curve analysis was carried out to assess the performance of the model by calculating the area under the curve (AUC). Statistical tests were performed using R 3.5.1 (www.r-project.org) and Prism-GraphPad software. One-way ANOVA was used for multiple comparisons, and *p* < 0.05 was considered statistically significant.

## Results

### Clinical characteristics of CTs, patients with SCLC and patients with NSCLC

In this study, we recruited 15 healthy individuals as the control (CT) group, 10 patients with early-stage SCLC as the SCLC group and 10 patients with early-stage NSCLC as the NSCLC group. Patients with SCLC were in the limited-stage and NSCLC were in stage I–II. TNM grading was used for all the lung cancer patients; clinical information about age, sex, smoking history, tumour stage and pathological types are summarized in Table 1. There were no differences in age, sex or smoking history among the three groups, and there were no differences in cancer stage between the SCLC and NSCLC groups.

**Table 1:** Clinical characterization of participants in the CT, SCLC, and NSCLC groups.

Characteristics	Screening				Verification				Total			
	CT group (*n* = 15)	SCLC group (*n* = 10)	NSCLC group (*n* = 10)	*p*	CT group (*n* = 9)	SCLC group (*n* = 15)	NSCLC group (*n* = 15)	*p*	CT group (*n* = 24)	SCLC group (*n* = 25)	NSCLC group (*n* = 25)	*p*
**Sex**				0.312				1.000				0.417
Male	7	8	5		7	11	12		14	19	17	
Female	8	2	5		2	4	3		10	6	8	
Age	66.1 ± 6.4	58.6 ± 7.3	63.2 ± 10.3	0.084	60.6 ± 8.4	63.7 ± 8.4	64.9 ± 6.0	0.401	64.0 ± 7.5	61.7 ± 8.2	64.2 ± 7.8	0.459
**Smoking history**				1.000				0.741				0.805
Yes	5	3	3		2	4	6		7	7	9	
No	10	7	7		7	11	9		17	18	16	
**Lung cancer TNM staging**												
T				0.628				1.000				0.758
1	–	6	8		–	11	10		–	17	18	
2	–	4	2		–	4	5		–	8	7	
N				1.000				1.000				0.747
0	–	3	2		–	4	3		–	7	6	
1	–	7	8		–	11	12		–	18	19	
**Stage**				1.000				1.000				0.508
I	–	3	2		-	4	3		–	7	5	
II	–	7	8		-	11	12		–	18	20	
**Pathology(NSCLC)**												
Adenocarcinoma	–	–	7	–	–	–	8	–	–	–	–	15
Squamous cell carcinoma	–	–	3	–	–	–	7	–	–	–	–	10

### Characterization of lung cancer patient plasma-derived sEV-enriched fractions by specific screening

**Figure 2:**
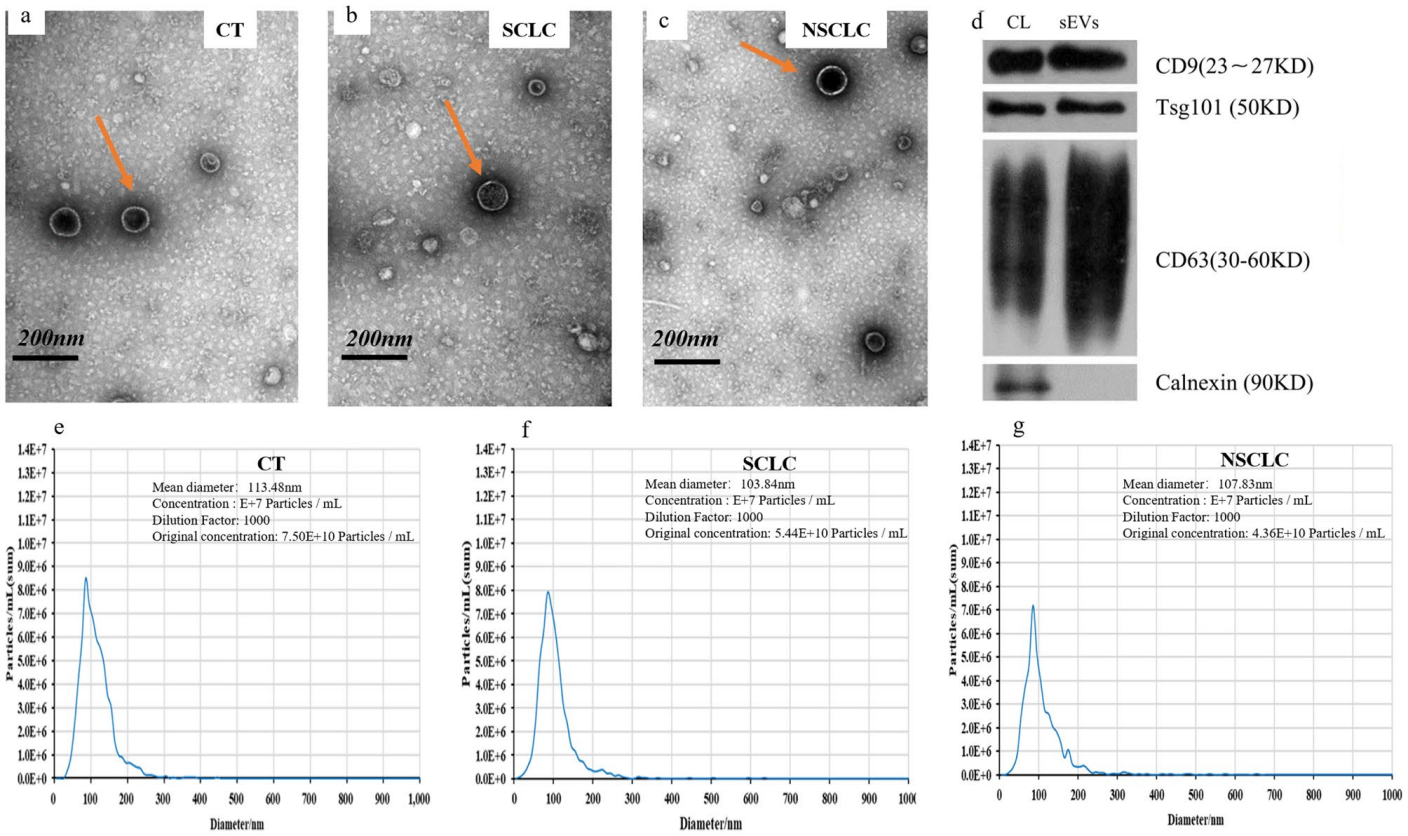
Isolated sEV-enriched fractions from participants’ plasma (**a**–**c**) TEM images showing that sEVs of three groups are oval or bowl-shaped capsules without a nucleus. d. The Western blot analysis of sEVs, markers CD9, CD63and TSG101were all detected in the sEV-enriched fractions isolated from plasma, and Calnexin, a negative marker of sEVs was absent in our isolated sEVs-enriched fraction samples. (**e**–**g**) NTA showed that the diameter and concentration of EVs for three groups. CL, control cell lysate.

We isolated sEVs from plasma samples of the CT, SCLC and NSCLC groups using SEC and characterized them by TEM, NTA and Western blotting. After fixation, adhesion, negative staining, and visualization, TEM images showed that sEVs were small cap-shaped membrane vesicles (Fig. 2a, b, c). The isolated sEVs were highly enriched in sEV-specific markers, such as CD9, CD63 and TSG101, whereas calnexin, a negative sEV marker, was not detected (Fig. 2d). In addition, NTA showed that the diameter and concentration of EVs for the three groups (Table S2, Fig. 2e, f, g).

### Comparison of miRNA profiling and bioinformatics analysis of sEV-derived miRNAs among the CT, SCLC and NSCLC groups

**Figure 3:**
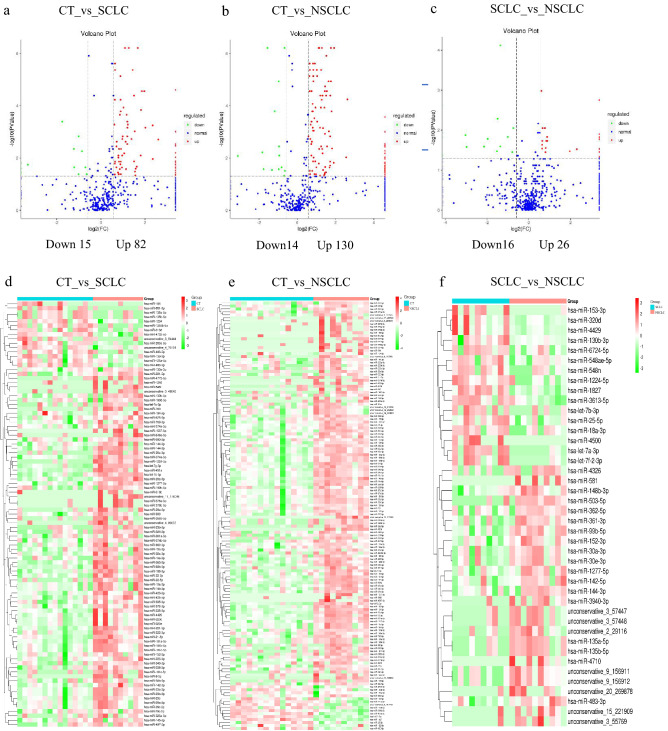
Identification and functional enrichment analysis of differentially expressed sEV-derived miRNAs. Volcano plot of differentially expressed sEV-derived miRNAs in CT versus SCLC, CT versus NSCLC and SCLS versus NSCLC (**a**–c). Each point represents an miRNA, red represents upregulated miRNA, green represents downregulated miRNA, and blue represents non-differentially expressed miRNAs. Heatmap of differentially expressed sEV miRNAs in CT versus SCLC, CT versus NSCLC and SCLS versus NSCLC (**d**–**f**). Red indicates high relative expression, and green indicates low relative expression.

Next-generation sequencing was performed on sEVs derived from participant plasma samples. This revealed the presence of a total of 1759 miRNAs, of which 1381 were known miRNAs. We included miRNAs with an average TPM > 10 for differential expression analysis to avoid bias that could be induced by miRNAs with relatively low expression levels. When the SCLC group was compared with the CT group, a total of 97 DEMs exhibited a > 1.5-fold change, with *p* < 0.05; of these DEMs, 82 were upregulated, and 15 were downregulated. In the comparison of the NSCLC group with the CT group, a total of 144 DEMs were identified, of which 130 were upregulated and 14 were downregulated. Finally, comparing the NSCLC group with the SCLC group, we identified a total of 42 DEMs, of which 26 were upregulated and 16 were downregulated. These data are depicted as volcano plots (Fig. 3a–c), and heatmaps provide a visual representation of the differences in the miRNA expression levels of the CT group versus the SCLC group, the CT group versus the NSCLC group and the SCLC group versus the NSCLC group (Fig. 3d–f).

**Figure 4:**
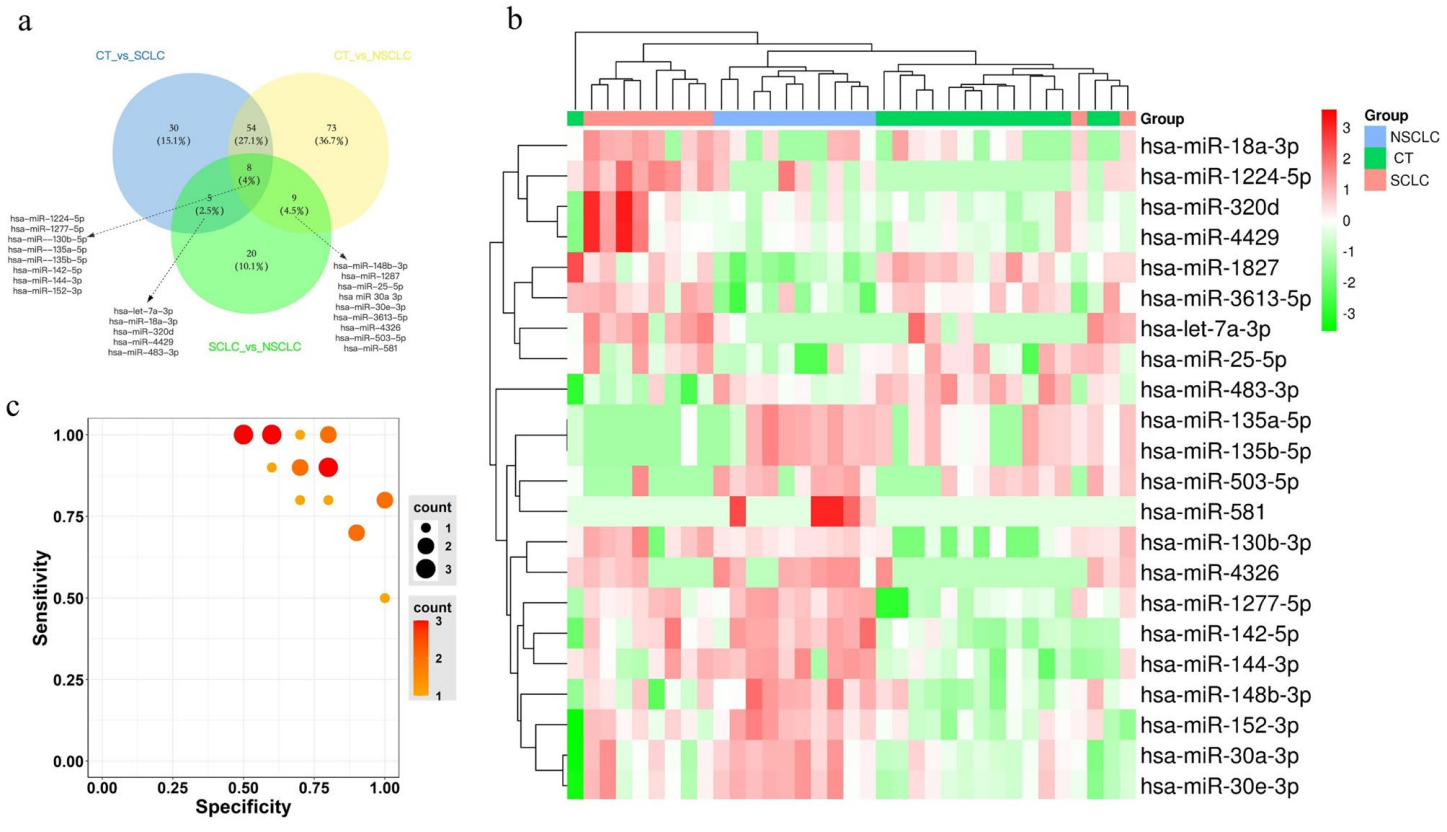
Plasma sEV-enriched fraction-derived miRNAs profiles that could be used to discriminate SCLC from NSCLC. (**a**) Venn diagram comparing the differential expression of sEV-derived miRNAs, each circle representing the number of differentially expressed sEV-derived miRNAs between two conditions. (**b**) Heat map of the 22 DEM expression levels across all 35 samples in our plasma sEV-enriched fraction miRNA dataset. (**c**) The specificity and sensitivity of each DEM for identifying SCLC versus NSCLC patients in our plasma sEV-enriched fraction miRNA dataset.

To identify sEV-derived miRNAs that could be used for SCLC/NSCLC identification, we constructed Venn diagrams (Fig. 4a). Among the 42 differentially expressed miRNAs between the SCLC group and the NSCLC group, 5 (let-7a-3p, miRNA-18a-3p, miRNA-320d, miRNA-4429, and miRNA-483-3p) were specific to SCLC, 9 (miRNA-148b-3p, miRNA-1827, miRNA-25-5p, miRNA-30a-3p, miRNA-30e-3p, miRNA-3613-5p, miRNA-4326, miRNA-503-5p, and miRNA-581) were specific to NSCLC, and 8 (miRNA-1224-5p, miRNA-1277-5p, miRNA-130b-3p, miRNA-135a-5p, miRNA-135b-5p, miRNA-142-5p, miRNA-144-3p, and miRNA-152-3p) could be used to discriminate the CT, SCLC and NSCLC groups. The expression levels of 22 DEMs across all 35 samples in the plasma-derived sEV-enriched fraction of the miRNA dataset are shown in the heatmap (Fig. 4b) and volcano plots (Fig. S1a-c). The specificity and sensitivity of the 22 DEMs for identifying SCLC patients versus NSCLC patients in our plasma-derived sEV-enriched fraction miRNA dataset are shown on the analytical diagram (Fig. 4c). We found that 13 miRNAs were significantly upregulated and 9 miRNAs were downregulated in NSCLC relative to SCLC (Table S3).

### Functional annotation and identification of differentially-expressed miRNAs

**Figure 5:**
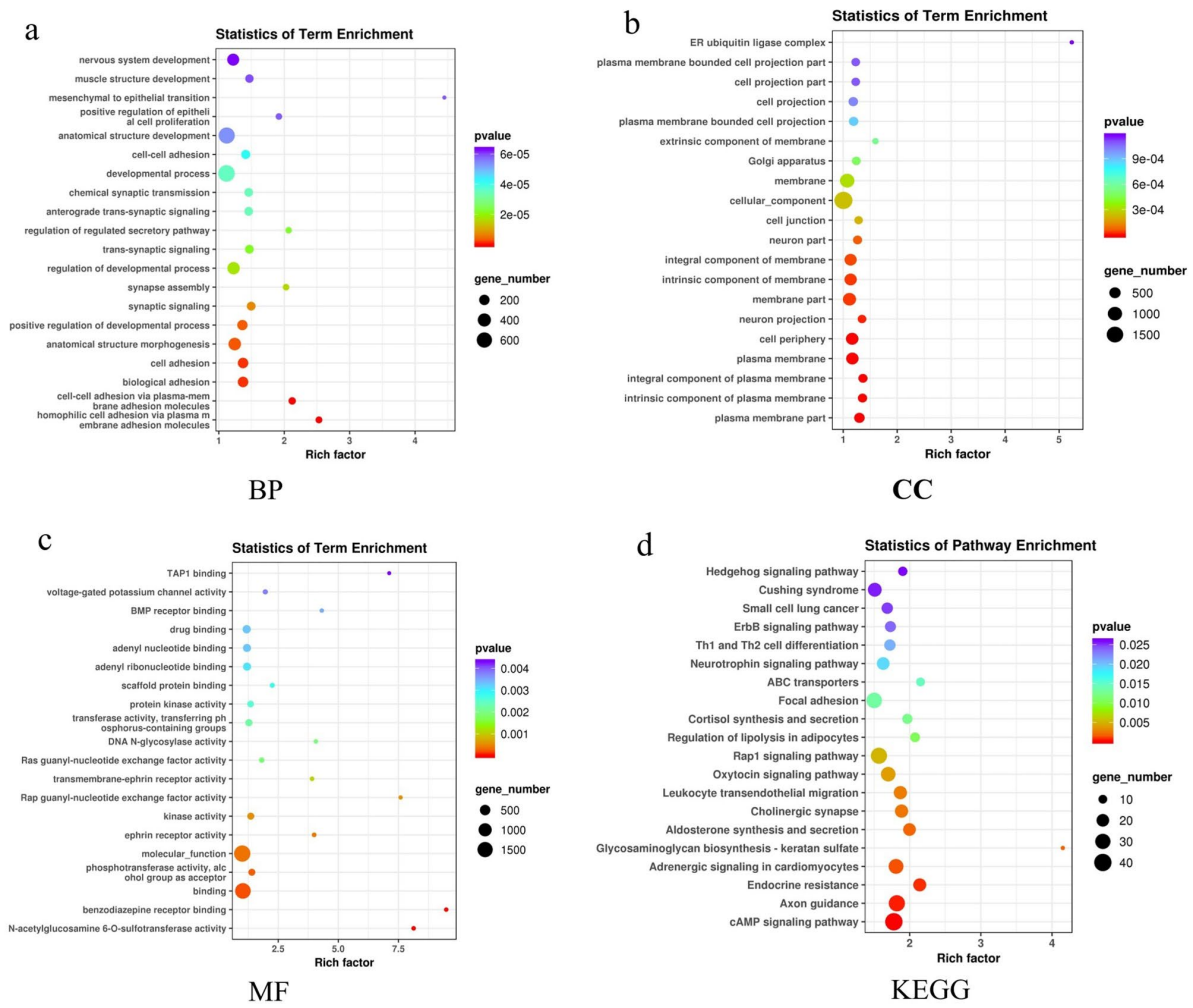
GO enrichment and KEGG pathway analysis of the 22 DEMs comparing SCLC and NSCLC. Advanced bubble chart shows enrichment of genes in signaling pathways. BP, biological processes; CC, cellular component; MF, molecular function.

We compared the biological characteristics of the 22 DEMs between the NSCLC and SCLC groups. The GO terms associated with the DEMs are shown in Fig. 5a–c. Regarding biological processes, the DEMs between the SCLC and NSCLC groups appeared to be most significantly enriched in homophilic cell adhesion via plasma membrane adhesion molecules and cell‒cell adhesion via plasma-membrane adhesion molecules, biological adhesion, positive regulation of developmental process, and regulation of regulated secretory pathways (*p* = 2.71E-09, *p* = 8.04E-05, *p* = 8.72E-09, *p* = 3.39E-06, *p* = 2.24E-05, respectively). For the cellular component, the most significantly enriched terms were plasma membrane, cell periphery, and intrinsic component of membrane (*p* = 5.03E-09, *p* = 3.66E-07, *p* = 2.47E-05, respectively). Finally, for molecular functions, the most significantly enriched terms were kinase activity, transferase activity, transferring phosphorus-containing groups and protein binding (*p* = 3.89 E-04, *p* = 5.42 E-04, *p* = 0.0022, respectively). The KEGG results indicated that the DEMs between the NSCLC and SCLC groups were clearly related to pathways associated with cAMP signalling, endocrine resistance, leukocyte transendothelial migration, Rap1 signalling and ErbB signalling (*p* = 1.96 E-04, *p* = 5.52E-04, *p* = 0.0027, *p* = 0.0051, *p* = 0.0237), as shown in Fig. 5d.

Furthermore, we applied GO and KEGG to analyse the DEMs between the CT group vs. the SCLC group, the CT group vs. the NSCLC group and the SCLC group vs. the NSCLC group. Comparing the SCLC group with the CT group revealed 97 DEMs. For biological processes, these DEMS appeared to be most significantly enriched in cell‒cell signalling and multicellular organism development (*p* = 1.15E-07, *p* = 1.61E-06); for cellular components, the most significantly enriched terms were plasma membrane and cell periphery (*p* = 2.36E-06, *p* = 2.62E-05); and for molecular function, the most significantly enriched terms were transcription regulatory region sequence-specific DNA binding and kinase activity (*p* = 3.07E-09, *p* = 5.15E-09) (Fig. S2a-c). KEGG analysis showed clear differences in the MAPK signalling and cGMP-PKG signalling pathways (*p* = 7.72E-06, *p* = 1.17E-04) (Fig. S2d). Next, we identified 144 DEMs between the NSCLC and CT groups; these DEMs were enriched in the biological processes of epithelium development and cell differentiation (*p* = 1.45E-07, *p* = 4.96E-07); for cellular components, the most significantly enriched terms were membrane and cytoplasmic part (*p* = 2.66E-06, *p* = 4.61E-06); and for molecular functions, the most significantly enriched terms were protein binding and growth factor binding (*p* = 1.56E-06, *p* = 5.48E-05) (Fig. S3a-c). KEGG analysis showed clear differences in the Ras signalling pathway and the Rap1 signalling pathway (*p* = 5.06E-04, *p* = 9.98 E-04) (Fig. S3d). Additionally, 42 DEMs were identified by comparing the NSCLC group with the SCLC group. In terms of biological processes, these DEMs appeared to be most significantly enriched in positive regulation of cellular process and multicellular organism development (*p* = 9.09E-11, *p* = 1.24E-08); for the cellular component, the most significantly enriched terms were plasma membrane part and cell junction (*p* = 5.16E-08, *p* = 5.29E-04); and for molecular function, the most significantly enriched terms were protein binding and kinase binding (*p* = 5.22E-07, *p* = 0.256) (Fig. S4a-c). The KEGG results indicated that DEMs were clearly different for the aldosterone synthesis and secretion pathways and the cAMP signalling pathway (*p* = 0.0140, *p* = 0.0390) (Fig. S4d).

### Weighted correlation network analysis (WGCNA)

**Figure 6:**
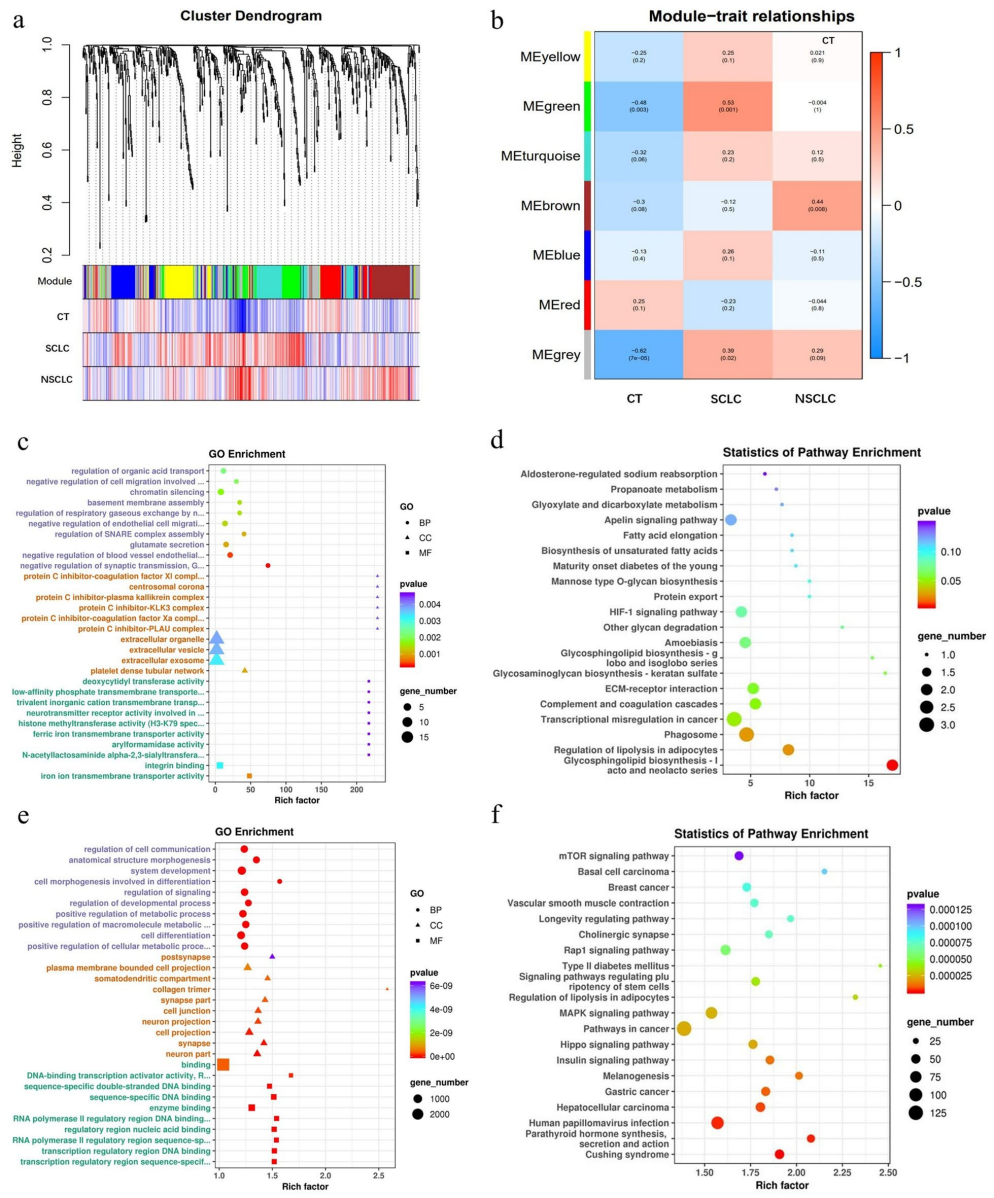
WGCNA network module mining, GO enrichment and KEGG pathway analysis of MEgreen and MEbrown. (**a**) The plasma-derived sEV miRNAs analyzed by the WGCNA method. (**b**) Module-pathological type relationships of consensus module eigengenes and different pathological types of lung cancer. Intensity and direction of correlations are indicated on the right side of the heatmap (red, positively correlated; blue, negatively correlated. (**c**, **d**) GO enrichment and KEGG pathway analysis of miRNAs in the MEgreen. (**e**, **f**) GO enrichment and KEGG pathway analysis of miRNAs in the MEbrown.

WGCNA with a soft-thresholding power value equal to 5 revealed the coexpression of genes/transcripts in a total of seven modules. Each module was assigned a unique colour label, namely, yellow, green, turquoise, brown, blue, red and grey, as shown in the cluster dendrogram (Fig. 6a). A matrix showing module trait relationships and corresponding *p* values between these seven identified modules of group traits is presented as a heatmap (Fig. 6b). Module eigengenes (MEs) were calculated and could represent each module. Among the seven modules, the MEs of module MEgreen (*r* = 0.53, *p* = 0.001) were positively correlated with the SCLC group, whereas those of module MEbrown were positively correlated with the NSCLC group (*r* = 0.44, *p* = 0.008).

In the module MEgreen, biological processes were most significantly enriched for negative regulation of synaptic transmission and negative regulation of cell migration involved in sprouting angiogenesis (*p* = 2.93E-04, *p* = 0.0020); for the cellular component, the most significantly enriched terms were platelet dense tubular network and extracellular exosome (*p* = 0.0010, *p* = 0.0032); and for molecular function, the most significantly enriched terms were iron ion transmembrane transporter activity, integrin binding and histone methyltransferase activity (*p* = 0.0007, *p* = 0.0032, *p* = 0.0046), as shown in Fig. 6c. The KEGG pathways that were most significantly enriched by the sEV miRNAs in the module MEgreen were phagosome and transcriptional misregulation in cancer (*p* = 0.0260, *p* = 0.0495), as shown in Fig. 6d. In the module MEbrown, biological processes were most significantly enriched in cell development and positive regulation of metabolic processes (*p* = 1.50E-12, *p* = 1.90E-11); for cellular components, the most significantly enriched terms were cell projection and plasma membrane bounded cell projection (*p* = 3.19E-11, *p* = 6.21E-10); and for molecular functions, the most significantly enriched terms were sequence-specific DNA binding, protein binding, and kinase binding (*p* = 3.44E-12, *p* = 6.02E-11, *p* = 9.48E-08), as shown in Fig. 6e. The KEGG pathways that were most significantly enriched by the sEV miRNAs in the module MEbrown were the Hippo signalling pathway, endocrine resistance, pathways in cancer, MAPK signalling pathway, and mTOR signalling pathway (*p* = 2.25E-05, *p* = 2.30E-05, *p* = 2.43E-05, *p* = 1.30 E-04), as shown in Fig. 6f.

### miRNA target gene prediction and qRT‒PCR validation

**Figure 7:**
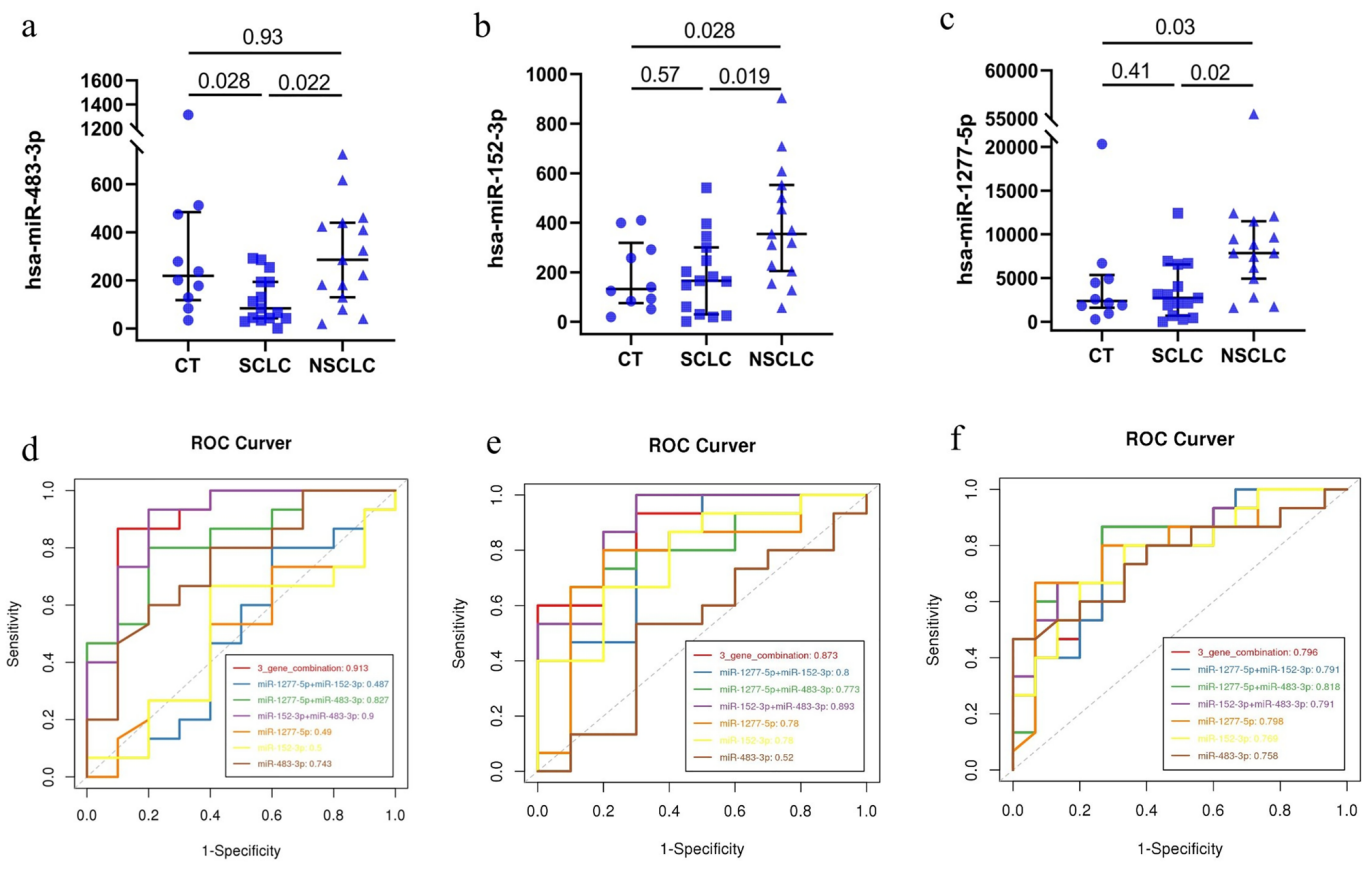
The expression levels and AUC of miR-483-3p, miR-152-3p, and miR-1277-5p of plasma-derived sEVs for validation. (**a**–**c**). The expression levels of miR-483-3p, miR-152-3p, and miR-1277-5p, respectively. The ordinate (y axis) is relative expression of miRNA. The abscissa of the upper edge is *P*-values, estimated by Mann–Whitney independent t-testing. (**d**–**f**) The AUC result of miR-483-3p, miR-152-3p, and miR-1277-5p from CT versus SCLC (**d**), CT versus NSCLC(**e**) , and SCLS versus NSCLC (**f**) , and combinations of two or three of these miRNAs.

Next, we recruited another 9 healthy individuals as controls, 15 early-stage SCLC patients and 15 early-stage NSCLC patients as the validation cohorts. The inclusion criteria were the same as those at the screening stage. The demographic and clinical information about the age, sex, smoking history, tumour stage and pathological types of the volunteers are summarized in Table 1. The differentially expressed sEV-derived miRNAs of the 39 volunteers in the CT, SCLC and NSCLC groups were verified. On the basis of the mode of screening results, we considered the expression values of six candidate sEV-derived miRNAs (miR-483-3p, miR-152-3p, miR-1277-5p, miR-130b-3p, miR-25-5p, and miR-4429) as a feature of subsequent analysis. The primer sequences used for qRT‒PCR are shown in Table S2. The expression level of miRNA-483-3p was found to be significantly downregulated in the SCLC group compared with the NSCLC group or the CT group (CT vs. SCLC, *p* = 0.0453; CT vs. NSCLC, *p* = 0.8200; SCLC vs. NSCLC, *p* = 0.0401) (Fig. 7a). The expression level of miRNA-152-3p was significantly upregulated in the NSCLC group compared with the SCLC group or the CT group (CT vs. SCLC, *p* = 0.8709; CT vs. NSCLC, *p* = 0.0103; SCLC vs. NSCLC, *p* = 0.0024) (Fig. 7b). Additionally, miRNA-1277-5p was significantly upregulated in the NSCLC group relative to the SCLC group and the control group (CT vs. SCLC, *p* = 0.8709; CT vs. NSCLC, *p* = 0.0103; SCLC vs. NSCLC, *p* = 0.0024) (Fig. 7c). The expression levels of miRNA-130b-3p was not significantly different among the CT, SCLC and NSCLC groups (CT vs. SCLC, *p* = 0.6020; CT vs. NSCLC, p = 0.1754; SCLC vs. NSCLC, p = 0.3296). However, the expression levels of miRNA-4429 and miRNA-25-5p were upregulated as NSCLC group compared with SCLC group, which were contrary to the screening result.

Furthermore, ROC curves were evaluated to calculate the diagnostic value of miRNA-483-3p, miRNA-152-3p and miRNA-1277-5p, and these ROC curves were compared between the NSCLC and SCLC groups. High specificity and sensitivity was demonstrated (miRNA-483-3p: AUC = 0.758, specificity = 0.8, sensitivity = 0.6; miRNA-152-3p: AUC = 0.769, specificity = 0.8, sensitivity = 0.667; miRNA-1277-5p: AUC = 0.798, specificity = 0.8, sensitivity = 0.733) (Fig. 7d–f, Table S3). Additionally, we analysed combinations of two or three of these miRNAs, and the high specificity and sensitivity for NSCLC vs. SCLC when combination of miR-152-3p and miR-1277-5p (AUC = 0.791, specificity = 0.867, sensitivity = 0.733) (Fig. 7d–f, Table S4).

### Functional roles of miRNA-483-3p, miRNA-152-3p and miRNA-1277-5p

Biological process and KEGG analyses were used to analyse the functional roles of miRNA-483-3p, miRNA-152-3p and miRNA-1277-5p (Fig. S6). For miRNA-483-3p, the related biological processes were mostly regulation of the regulated secretory pathway, substrate adhesion-dependent cell spreading and cellular response to chemical stimulus (*p* = 2.23E-05, *p* = 1.02E-04, *p* = 0.0051). KEGG pathways related to miRNA-483-3p were mainly cAMP signalling, aldosterone synthesis and secretion, and cGMP-PKG signalling (*p* = 1.97E-04, *p* = 0.0018, *p* = 0.0337). For miRNA-152-3p, the related biological processes were mostly cell adhesion, anatomical structure morphogenesis and developmental process (*p* = 1.12E-06, *p* = 3.04E-06, *p* = 3.55E-05). Finally, for miRNA-1277-5p, the related biological processes were mostly protein maturation and protein metabolic processes (*p* = 0.05, *p* = 0.05).

## Discussion

Lung cancer remains one of the most threatening malignancies with high morbidity and mortality rates. SCLC is a high-grade neuroendocrine lung cancer that accounts for approximately 15% of all lung cancer cases, and is strongly associated with severe tobacco exposure. SCLC is characterized by high proliferation, high circulating tumour cell burden, early and extensive metastasis, high mortality, and poor prognosis^30–32^. The survival time of patients with SCLC is approximately ten months, and the five-year overall survival rate is 6%^33^. NSCLC accounts for approximately 80% of the total number of lung cancer cases; the growth, division and proliferation of NSCLC are slow, and it is characterized by late metastasis and spread. Despite the continuous improvement in the diagnosis and treatment of NSCLC, its 5-year survival rate is still low^34^. The incidence, metastasis, treatment and prognosis of the two pathological types of lung cancer are completely different. In practice, it may be difficult to make a pathological diagnosis in some early lung cancer patients, which often delays treatment. Hence, biomarkers that can be used for the diagnosis of early SCLC and NSCLC have attracted considerable attention.

Recently, sEVs have attracted substantial attention as a new minimally invasive diagnostic tool to identify asymptomatic cancer patients^15,16^. The cargos of sEVs isolated from plasma include many different types of cell-free nucleic acids and proteins; among these cargos, miRNAs have been a focus of biomarker-related translational studies because of their abundance and stability^19,20^. Many studies have confirmed that miRNAs can be potential biomarkers for lung cancer^35,36^, but there are no reports on the use of miRNAs from plasma sEVs for the differential diagnosis of SCLC and NSCLC. In the present study, sEVs were isolated by SEC methods and characterized by TEM, NTA and Western blotting. NGS was then performed on 35 samples of miRNAs derived from sEVs, and their expression levels were compared; DEMs among the CT, SCLC and NSCLC groups were identified.

Here, GO and KEGG analyses were conducted with the DEMs of the three groups. There were 97 DEMs between the SCLC and CT groups, and biological process enrichment in cellular components and potential pathways MAPK signalling and cGMP-PKG signalling were observed. Next, we identified 144 DEMs between the NSCLC and CT groups, and for biological processes, the DEMs were enriched in epithelium development and cell differentiation, and KEGG analysis revealed associations with the Ras signalling pathway and the Rap1 signalling pathway. The MAPK signalling, cGMP-PKG signalling, Ras signalling and Rap1 signalling pathways were already reported to be important pathways for the occurrence and growth of cancer^37–40^. The functional enrichment of DEMs between different groups was significantly different. Furthermore, we compared the differences in miRNAs in the CT, SCLC and NSCLC groups and found that 22 DEMs had the potential to act as biomarkers for the differential diagnosis of SCLC and NSCLC. The results showed different biological processes of cell‒cell signalling and multicellular organism development and pathways of MAPK signalling, cGMP-PKG signalling, TGF-beta signalling and Wnt signalling, which also suggests differences in the biological functions of these DEMs between the SCLC and NSCLC groups. All these analyses were helpful in further identifying the target miRNAs in early SCLC and early NSCLC.

WGCNA can be used to identify cluster modules of highly related genes^41,42^. We constructed a regulatory network by using differentially expressed sEV miRNAs between SCLC and NSCLC to explore their association and possible mechanisms related to cancer occurrence and growth. We found that the module MEgreen was positively correlated with SCLC and applied GO and KEGG to analyse the miRNAs in this module. This revealed that the function of the cluster of genes enriched in the process of cell migration was involved in sprouting angiogenesis, iron ion transmembrane transporter activity, integrin binding and histone methyltransferase activity. Thus, it is confirmed that the function of this module may be related to the occurrence of early-stage SCLC. The module MEbrown was correlated with NSCLC, with the clustered genes enriched in the processes of cell projection, cell junction and plasma membrane bounded cell projection, and the Hippo signalling pathway, endocrine resistance and pathways in cancer; these results provided information about the occurrence and growth of early-stage NSCLC. This analysis also suggests the presence of different biological functions between NSCLC and SCLC and will be helpful for selecting target miRNAs.

Based on these screening results and reviewing previous published literature about cancer-related markers, we finally identified 6 DEMs for further verification, namely, miR-4429, miR-483-3p, miR-130b-3p, miR-152-3p, hsa-miR-25-5p and miR-1277-5p. Additionally, we recruited further healthy individuals, early-stage SCLC patients and early-stage NSCLC patients for this verification step. By measuring the expression levels of plasma-derived sEV miRNAs and analysing ROC curves, we confirmed plasma-derived sEV miR-483-3p as a biomarker for the early diagnosis of early-stage SCLC and miR-152-3p and miR-1277-5p as biomarkers for the early diagnosis of NSCLC.

MiRNA-483-3p is a key regulator of gastric cancer, colorectal cancer, breast cancer, and hepatocellular carcinomas^41–44^ and can suppress cancer growth, cancer cell apoptosis, metastasis, and cancer recurrence^42,44,45^. However, the role of plasma-derived sEV miRNA-483-3p in early lung cancer has not been reported. In the present study, we found that this miRNA was downregulated in the SCLC group compared with the CT and NSCLC groups and had significant specificity and sensitivity. GO enrichment analysis indicated a biological function of miRNA-483-3p in cell adhesion and the regulation of the regulated secretory pathway, and KEGG pathway analysis suggested its potential involvement in the cAMP signalling pathway, endocrine resistance, and aldosterone synthesis and secretion. Its biological function also confirmed that SCLC is a tumour related to neuroendocrine function, which is consistent with recent reports^46^.

MiRNA-152-3p is a member of the miR-148/152 family, which includes miR-148a, miR-148b, and miR-152-3p with relatively conserved sequences^47^. Studies on miR-152-3p have focused on its roles in tumour suppression or promotion^48,49^. Many studies have suggested that miR-152-3p is involved in the development of gastric cancer, colorectal cancer, breast cancer, liver cancer and prostate cancer^48–52^, and research has also focused on the role of miR-152-3p in inhibiting cancer or promoting cancer. Some studies suggest that miRNA-152-3p is related to the regulation of the long noncoding RNA, chaperonin-containing TCP1 subunit 6A (CCT6A) pathway, and phosphatase and tensin homologue (PTEN)^53,54^. According to most recent publications, there are no reports of correlations between plasma-derived sEV miR-152-3p and early-stage lung cancer. In the present study, we found that this miRNA was upregulated in the NSCLC group compared with the CT and SCLC groups and had significant specificity and sensitivity. GO enrichment analysis indicated that miR-152-3p performs biological functions related to cell adhesion, signal transduction, and regulation of signal transduction by p53. These biological processes are related to the occurrence and growth of many cancers^55,56^ and are consistent with the results of GO, KEGG and WGCNA in the screening stage.

According to available data, there are some studies on correlations of miRNA-1277-5p, which report that miRNA-1277-5p is downregulated in gastric cancer and inflammatory and carotid atherosclerosis^57–59^, but there are no reports about miRNA-1277-5p in lung cancer. In the present study, we found that the expression level of plasma-derived sEV miRNA-1277-5p was upregulated in NSCLC. At present, this report on miRNA-1277-5p appears to be unique, and this molecule may be a potential new target for the diagnosis of early-stage NSCLC.

This study has several limitations. First, the number of patients who participated in the study was relatively low. The data might be more convincing if the sample size could be increased. However, early lung cancer is mostly asymptomatic, and the diagnosis rate is low, so few patients were enrolled. Second, the biological characteristics of SCLC and NSCLC are complex, so we need to analyse their biological characteristics in greater detail to find the most significant differences between them and reveal the role of miRNAs in signalling pathways in future studies.

## Conclusions

Our study helps to advance the understanding of tumorigenesis of early-stage SCLC and early-stage NSCLC. This study also confirms that miRNA-483-3p derived from plasma sEVs can be used as a potential biomarker for the early diagnosis of SCLC, miRNA-152-3p and miRNA-1277-5p can be used as a potential biomarker for the early diagnosis of NSCLC respectively.

## Supplementary Information

Supplementary Information.

## Data Availability

The datasets generated and analysed during the current study are available in the National Genomics Data Center repository, OMIX001751 (https://ngdc.cncb.ac.cn/omix/preview/cnbnN32C) .
