# Supplementary material for: Evaluation of circulating small extracellular vesicle-derived miRNAs as diagnostic biomarkers for differentiating between different pathological types of early lung cancer
Source: Sci Rep. 2022 Oct 13;12:17201. doi: 10.1038/s41598-022-22194-0 (PMC9561663; doi:10.1038/s41598-022-22194-0)
Supplement: Supplementary file 1 — Supplementary Information. [file 41598_2022_22194_MOESM1_ESM.docx]

**Supplementary information**

| **MiRNA** | **Primer sequences** | |
| --- | --- | --- |
|  | **Forward (5'--3')** | **Reverse (5'--3')** |
| U6 | CTCGCTTCGGCAGCACA | AACGCTTCACGAATTTGCGT |
| miR-483-3p | CGCTCACTCCTCTCCTCC | GTCGTATCCAGTGCAGGGTCCGAGGTATTCGCACTGGATACGACAAGACG |
| miR-152-3p | GGCTCAGTGCATGACAGA | GTCGTATCCAGTGCAGGGTCCGAGGTATTCGCACTGGATACGACCCAAGT |
| miR-1277-5p | CGCCGCAAATATAGATAGATATGTACG | GTCGTATCCAGTGCAGGGTCCGAGGTATTCGCACTGGATACGACATACGTAC |
| miR-130b-3p | TCGCACTGGATACGACATGCCCT | CGCCAGTGCAATGATGAAA |
| miR-25-3p | ACGAGGCGGAGACTTGG | GTCGTATCCAGTGCAGGGTCCGAGGTATTCGCACTGGATACGACCAATTG |
| miR-4429 | CACGAAAAGCTGGGCTGA | GTCGTATCCAGTGCAGGGTCCGAGGTATTCGCACTGGATACGACCGCCTC |

**Table S1.** Primer sequences for qRT-PCR.

| **Item** | **Screening** | | | | **Verification** | | | |
| --- | --- | --- | --- | --- | --- | --- | --- | --- |
|  | **CT group** | **SCLC group** | **NSCLC group** | **P** | **CT group** | **SCLC group** | **NSCLC group** | **P** |
| Diameter(nm) | 110.47±6.02 | 108.19±7.07 | 108.65±6.01 | 0.634 | 111.01±7.21 | 108.30±7.12 | 110.13±6.46 | 0.611 |
| Concentration  (Particles / mL E+10) | 6.05±1.65 | 6.58±1.76 | 5.76±2.33 | 0.682 | 5.6±2.65 | 6.21±1.83 | 5.30±2.23 | 0.528 |

**Table S2.** The diameter and concentration of EVs for CT, SCLC and NSCLC groups

| **MiRNA** | **Accuracy** | **NPV** | **SN** | **SP** | **AUC** | **Regulated** |
| --- | --- | --- | --- | --- | --- | --- |
| miR-1827 | 0.90 | 1.000 | 1.0 | 0.8 | 0.97 | down |
| miR-135a-5p | 0.90 | 0.833 | 0.8 | 1.0 | 0.91 | up |
| miR-135b-5p | 0.90 | 0.833 | 0.8 | 1,0 | 0.91 | up |
| let-7a-3p | 0.90 | 1.000 | 1.0 | 0.8 | 0.88 | down |
| miR-3613-5p | 0.85 | 0.888 | 0.9 | 0.8 | 0.86 | down |
| miR-4429 | 0.80 | 1.000 | 1.0 | 0.6 | 0.84 | down |
| miR-483-3p | 0.80 | 1.000 | 1.0 | 0.6 | 0.84 | up |
| miR-148b-3p | 0.75 | 0.777 | 0.8 | 0.7 | 0.84 | up |
| miR-1224-5p | 0.80 | 0.750 | 0.7 | 0.9 | 0.83 | up |
| miR-18a-3p | 0.75 | 1.000 | 1.0 | 0.7 | 0.82 | down |
| miR-130b-3p | 0.85 | 1.000 | 1.0 | 0.7 | 0.82 | down |
| miR-30e-3p | 0.80 | 0.875 | 0.9 | 0.7 | 0.82 | up |
| miR-503-5p | 0.85 | 0.888 | 0.9 | 0.8 | 0.82 | up |
| miR-144-3p | 0.85 | 0.888 | 0.9 | 0.8 | 0.81 | up |
| miR-152-3p | 0.75 | 0.857 | 0.9 | 0.6 | 0.81 | down |
| miR-30a-3p | 0.80 | 1.000 | 1.0 | 0.6 | 0.81 | up |
| miR-142-5p | 0.80 | 0.875 | 0.9 | 0.7 | 0.80 | up |
| miR-25-5p | 0.75 | 1.000 | 1.0 | 0.5 | 0.80 | down |
| miR-320d | 0.75 | 1.000 | 1.0 | 0.5 | 0.78 | down |
| miR-1277-5p | 0.80 | 0.800 | 0.8 | 0.8 | 0.78 | up |
| miR-4326 | 0.80 | 0.750 | 0.7 | 0.9 | 0.78 | up |
| miR-581 | 0.75 | 0.666 | 0.5 | 1.0 | 0.75 | up |

**Table S3.** Assessment scale for the selection of differential diagnostic biomarker candidates from 22 DEMs which were compared between NSCLC with SCLC. Abbreviation: SN, sensitivity; SP, specificity;NPV, negative predictive value.

| **MiRNA** | **CT vs SCLC** | | | **CT vs NSCLC** | | | **SCLC vs NSCLC** | | |
| --- | --- | --- | --- | --- | --- | --- | --- | --- | --- |
|  | **SN** | **SP** | **AUC** | **SN** | **SP** | **AUC** | **SN** | **SP** | **AUC** |
| MiR-1277-5p | 0.27 | 0.60 | 0.490 | 0.80 | 0.80 | 0.780 | 0.80 | 0.73 | 0.798 |
| MiR-152-3p | 0.67 | 0.60 | 0.500 | 0.87 | 0.60 | 0.780 | 0.80 | 0.67 | 0.769 |
| MiR-483-3p | 0.80 | 0.60 | 0.743 | 0.27 | 0.60 | 0.520 | 0.80 | 0.60 | 0.758 |
| MiR-1277-5p+  miR-152-3p | 0.20(0.60~1.00) | 0.60(0.10~0.70) | 0.487 | 1.00(1.00~1.00) | 0.50(0.20~0.80) | 0.800 | 0.87(0.67~1.00) | 0.73(0.47~0.93) | 0.791 |
| MiR-1277-5p+  miR-483-3p | 0.80(0.60~1.00) | 0.80(0.50~1.00) | 0.827 | 0.80(0.60~1.00) | 0.70(0.40~1.00) | 0.773 | 0.87(0.67~1) | 0.73(0.47~0.93) | 0.818 |
| MiR-152-3p+  miR-483-3p | 0.93(0.80~1.00) | 0.80(0.50~1.00) | 0.900 | 1.00(1.00~1.00) | 0.70(0.40~1.00) | 0.893 | 0.67(0.40~0.87) | 0.87(0.667~1) | 0.791 |
| MiR-1277-5p+miR-152-3p+miR-483-3p | 0.87(0.67~1.00) | 0.90(0.70~1.00) | 0.913 | 1(1~1) | 0.50(0.20~0.80) | 0.800 | 0.87(0.73~1.00) | 0.73(0.47~0.93) | 0.796 |

**Table S4.** Comparison of the AUC, sensitivity and specifcity of miR-483-3p, miR-152-3p, and miR-1277-5p of plasma-derived sEVs for validation. Abbreviation: SN, sensitivity; SP, specifcity.


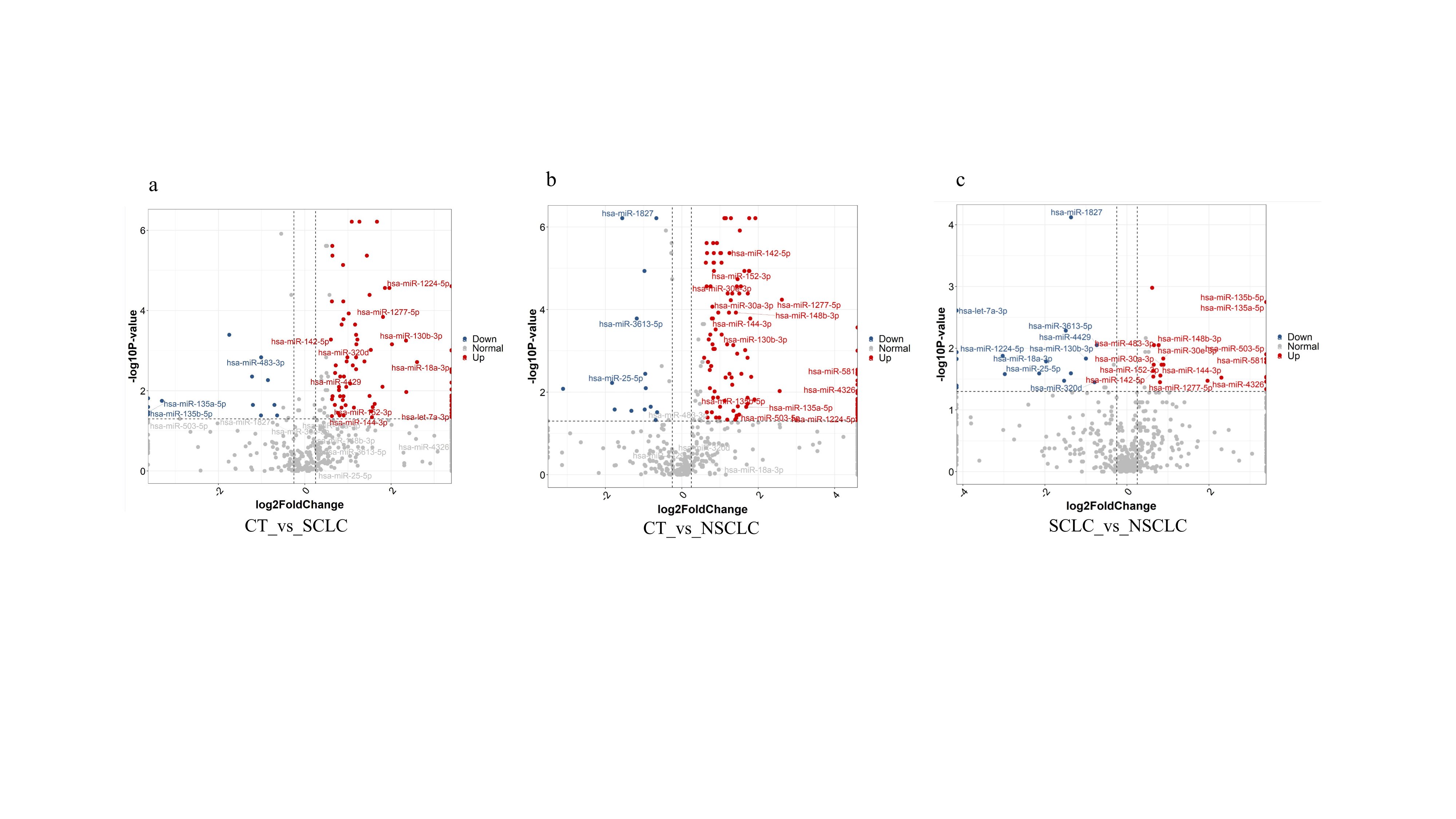


**Figure S1.** Volcano plot of 22 sEV-derived DEMs for CT versus SCLC, CT versus NSCLC and SCLC versus NSCLC. Each point represents a miRNA, red represents upregulated miRNA, blue represents downregulated miRNA, and black represents nondifferentially expressed miRNAs.


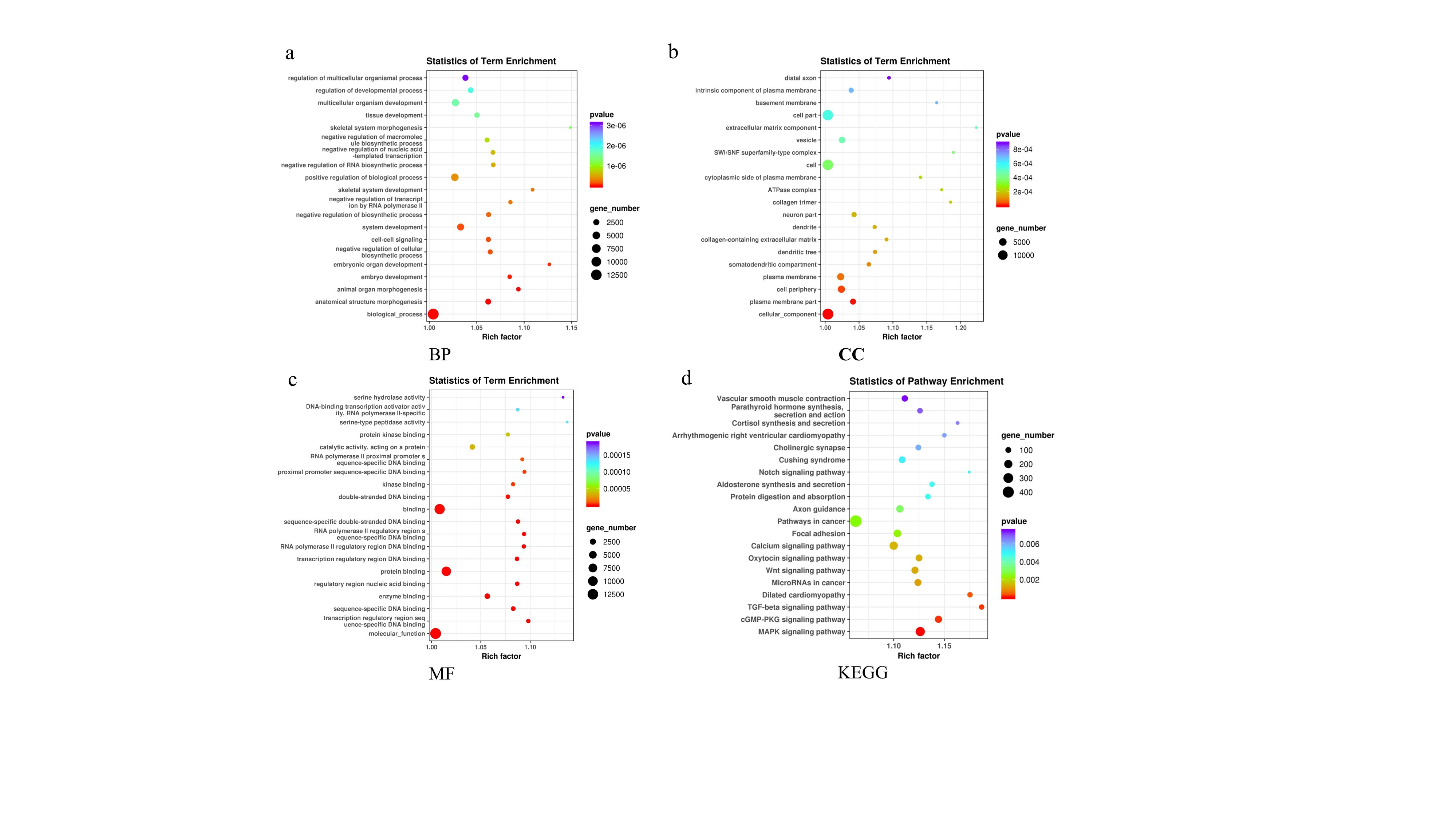


**Figures S2.** GO enrichment and KEGG pathway analysis of 97 DEMs between the CT and SCLC groups. GO enrichment for biological processes, cellular components and molecular function between CT and SCLC for DEMs are shown in Figures S2a-c. KEGG pathway of DEMs are shown in Figures S2d.


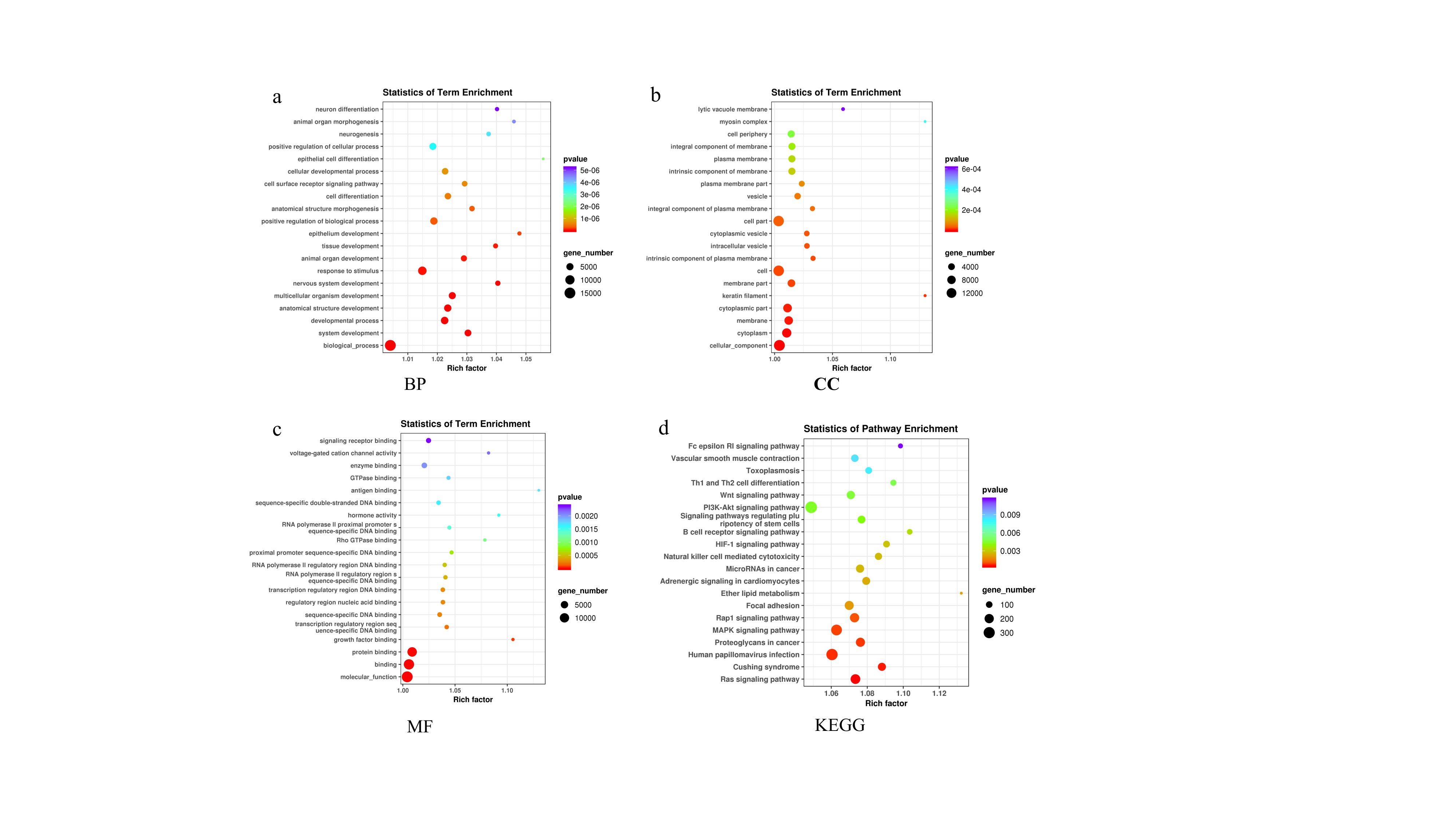


**Figures** **S3.** GO enrichment and KEGG pathway analysis of 144 DEMs between the CT and NSCLC groups. GO enrichment for biological processes, cellular components and molecular function of the DEMs are shown in Figures S3a-c. KEGG pathway of the DEMs are shown in Figure S3d.


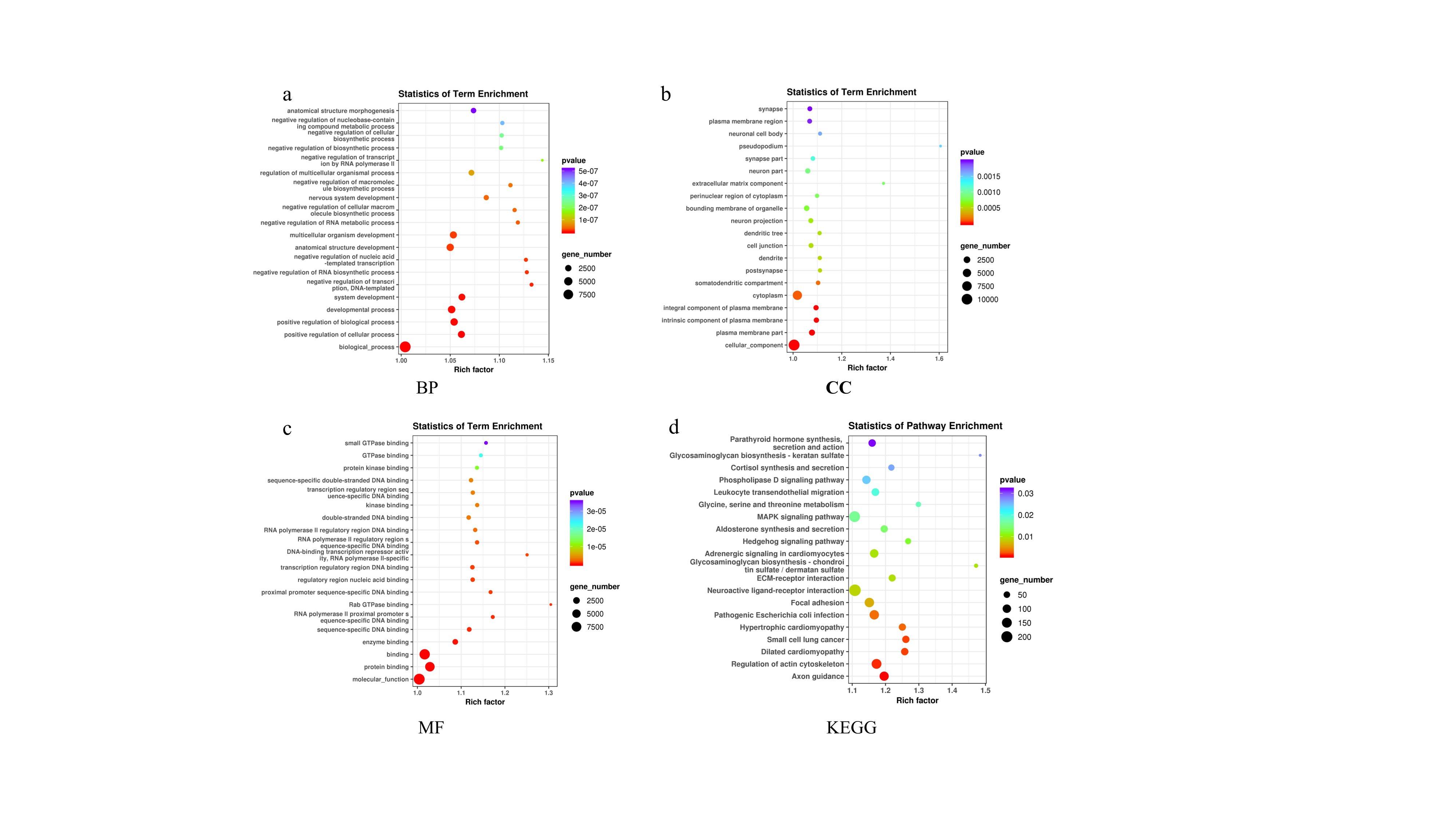


**Figures S4.** GO enrichment and KEGG pathway analysis of 42 DEMs between the SCLC and NSCLC groups. GO enrichment for biological processes, cellular components and molecular function of the DEMs are shown in Figures S 4 a-c. KEGG pathway of the DEMs are shown in Figures S 4 d.


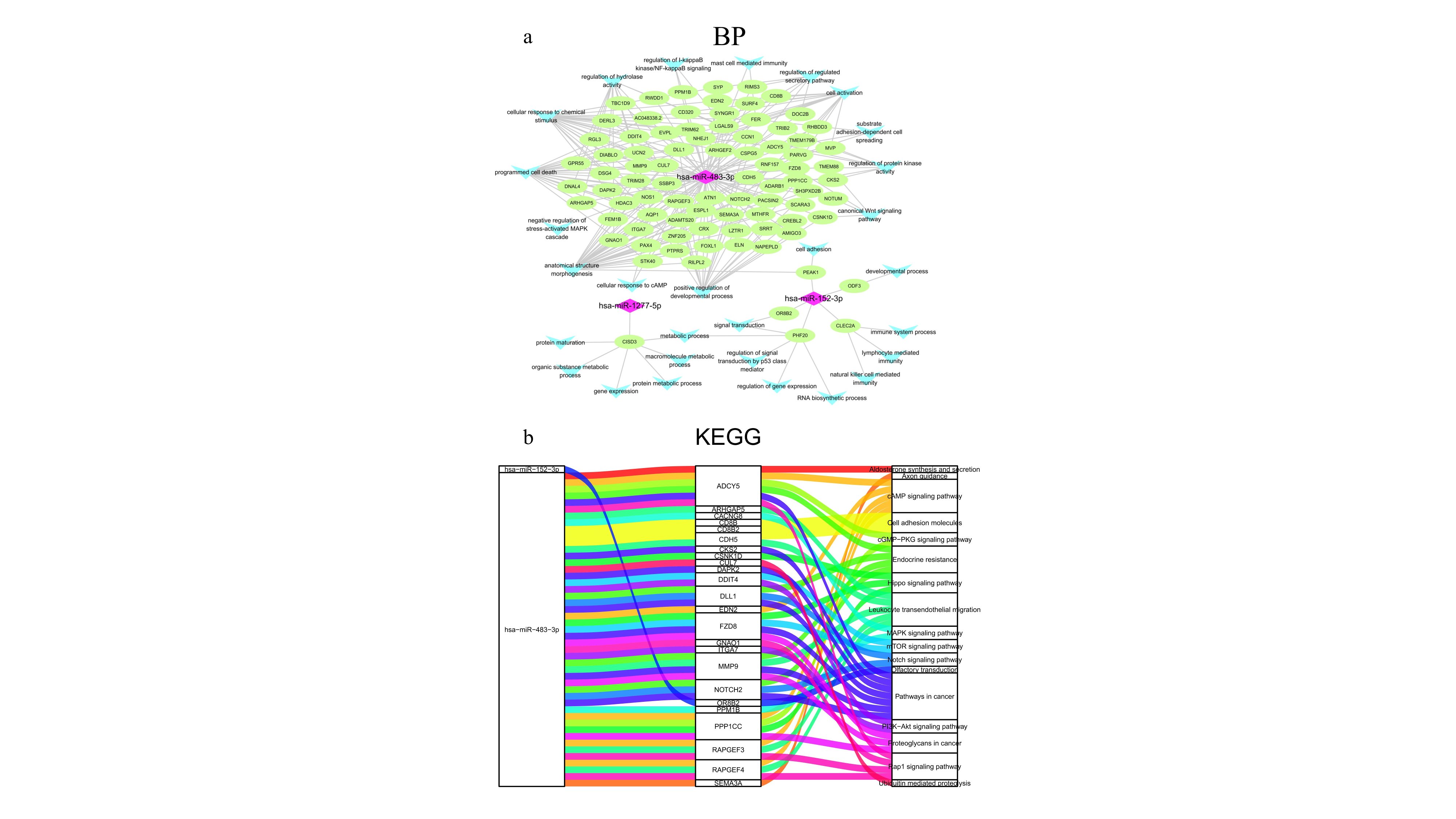


**Figure S5.** Biological process (BP) and KEGG analysis of miRNA-483-3p, miRNA-152-3p and miRNA-1277.
